# Comparison of Repeated Doses of C-kit-Positive Cardiac Cells versus a Single Equivalent Combined Dose in a Murine Model of Chronic Ischemic Cardiomyopathy

**DOI:** 10.3390/ijms22063145

**Published:** 2021-03-19

**Authors:** Qianhong Li, Yiru Guo, Yibing Nong, Alex Tomlin, Anna Gumpert, Xiaoping Zhu, Syed Adeel Hassan, Roberto Bolli

**Affiliations:** Division of Cardiovascular Medicine, Institute of Molecular Cardiology, University of Louisville, Louisville, KY 40292, USA; qhli0001@louisville.edu (Q.L.); yrguo@louisville.edu (Y.G.); yibing.nong@louisville.edu (Y.N.); alex.tomlin@louisville.edu (A.T.); anna.gumpert@louisville.edu (A.G.); xiaoping.zhu@louisville.edu (X.Z.); syedadeel.hassan@louisville.edu (S.A.H.)

**Keywords:** c-kit-positive cardiac cells, multiple doses, cell therapy, chronic ischemic cardiomyopathy, reperfused myocardial infarction

## Abstract

Using a murine model of chronic ischemic cardiomyopathy caused by an old myocardial infarction (MI), we have previously found that three doses of 1 × 10^6^ c-kit positive cardiac cells (CPCs) are more effective than a single dose of 1 × 10^6^ cells. The goal of this study was to determine whether the beneficial effects of three doses of CPCs (1 × 10^6^ cells each) can be fully replicated by a single combined dose of 3 × 10^6^ CPCs. Mice underwent a 60-min coronary occlusion; after 90 days of reperfusion, they received three echo-guided intraventricular infusions at 5-week intervals: (1) vehicle × 3; (2) one combined dose of CPCs (3 × 10^6^) and vehicle × 2; or (3) three doses of CPCs (1 × 10^6^ each). In the combined-dose group, left ventricular ejection fraction (LVEF) improved after the 1st CPC infusion, but not after the 2nd and 3rd (vehicle) infusions. In contrast, in the multiple-dose group, LVEF increased after each CPC infusion; at the final echo, LVEF averaged 35.2 ± 0.6% (*p* < 0.001 vs. the vehicle group, 27.3 ± 0.2%). At the end of the study, the total cumulative change in EF from pretreatment values was numerically greater in the multiple-dose group (6.6 ± 0.6%) than in the combined-dose group (4.8 ± 0.8%), although the difference was not statistically significant (*p* = 0.08). Hemodynamic studies showed that several parameters of LV function in the multiple-dose group were numerically greater than in the combined-dose group (*p* = 0.08 for the difference in LVEF). Compared with vehicle, cardiomyocyte cross-sectional area was reduced only in the multiple-dose group (−32.7%, 182.6 ± 15.1 µm^2^ vs. 271.5 ± 27.2 µm^2^, *p* < 0.05, in the risk region and −28.5%, 148.5 ± 12.1 µm^2^ vs. 207.6 ± 20.5 µm^2^, *p* < 0.05, in the noninfarcted region). LV weight/body weight ratio and LV weight/tibia length ratios were significantly reduced in both cell treated groups vs. the vehicle group, indicating the attenuation of LV hypertrophy; however, the lung weight/body weight ratio was significantly reduced only in the multiple-dose group, suggesting decreased pulmonary congestion. Taken together, these results indicate that in mice with chronic ischemic cardiomyopathy, the beneficial effects of three doses of CPCs on LV function and hypertrophy cannot be fully replicated with a single dose, notwithstanding the fact that the total number of cells delivered with one or three doses is the same. Thus, it is the multiplicity of doses, and not the total number of cells, that accounts for the superiority of the repeated-dose paradigm. This study supports the idea that the efficacy of cell therapy in heart failure can be augmented by repeated administrations.

## 1. Introduction

Cell therapy is a promising approach to heart failure, both in experimental animals and in humans [1]. One of the unresolved issues in this field is the optimal number of treatments. Traditionally, almost all preclinical and essentially all clinical studies have used one dose of cells [2,3]. However, it is now well recognized that after cell transplantation, the exogenous cells, whether adult or embryonic, do not engraft in the heart and disappear almost completely after a few weeks [3,4,5,6]. Consequently, it seems logical to modify traditional treatment protocols by administering repeated doses of cells in order to prolong their actions. Indeed, in recent years mounting evidence has suggested that repeated cell injections are more efficacious than a single injection [2,5,7,8,9]. Specifically, using models of chronic ischemic cardiomyopathy (old myocardial infarction [MI]), we found that three repeated doses of c-kit-positive cardiac cells (CPCs) in mice [7] and rats [8,9] and cardiac mesenchymal cells (CMCs) in mice [5] improve left ventricular (LV) function to a greater extent than a single dose; the fact that similar outcomes were observed with two different cell types and in two different species suggests that the greater efficacy of multiple doses is not dependent on species or cell type. Based on these observations and other studies, the concept is emerging that the efficacy of cell therapy in heart failure can be enhanced by the use of multiple doses [2,3].

One limitation of our previous studies [5,7,8,9] was that we compared two treatment strategies that resulted in different total numbers of cells being administered; that is, in the multiple-dose groups, the total number of cells transplanted was three times greater than in the single-dose groups. Because of this experimental design, one could argue that the therapeutic superiority of repeated doses was caused by the greater total number of cells transplanted rather than by the use of repeated injections per se. In other words, it was not the number of cell injections that caused greater efficacy but the total number of cells given. If this is the case, simply increasing the number of cells administered in one injection should achieve the same therapeutic effect as repeated injections.

Accordingly, in the present study, we compared the effects of the same cell number administered as a single dose or split into three doses. Specifically, we tested whether the improvement in LV function and hypertrophy produced by three doses of 1 × 10^6^ CPCs is greater than that of one large combined dose of 3 × 10^6^ CPCs. To emulate the common clinical setting of chronic ischemic cardiomyopathy, we used a mouse model of LV dysfunctions caused by a chronic MI. Unlike our previous study [7] and most previous mouse studies, we started cell therapy at 3 months after MI, when scar formation is complete and cardiac function is relatively stable.

## 2. Results

### 2.1. Exclusions and Gross Measurements

Of the 110 mice operated upon for surgical induction of MI, 21 (20%) died in the first 2 days. The remaining 89 mice were allowed to recover for 3 months and then echocardiographic studies were done to assess the severity of LV dysfunction. To ensure that only animals with severe LV dysfunction were analyzed, 29 mice with LVEF >30% were excluded. The remaining 60 mice received one of three treatments (vehicle, single combined CPC dose, or multiple CPC doses). Five mice died after the 1st injection, 2 after the 2nd injection, and 1 after the 3rd injection, all within 24 h of injection. The major cause of death was bleeding (Supplementary Table S1); this happened especially at the beginning of the study. 

Thus, a total of 52 mice were included in the final analysis: 18 in the vehicle group, 18 in the combined-dose group, and 16 in the multiple-dose group (Supplementary Table S1).

By experimental design, which was the same as our previous studies [5,7,10], body temperature was monitored and maintained between 36.8–37.2 °C in all groups during all coronary occlusion/reperfusion, echo-guided injection, echocardiographic imaging, and hemodynamic monitoring procedures (Supplementary Table S2A–C). Heart rate was also similar in all three groups (Supplementary Table S2). 

We measured body weight before each major procedure (Supplementary Table S3). In the vehicle group, there was a weight loss (−1.5 ± 0.8 g vs. pretreatment values) at the end of the 15-week follow-up; in contrast, in the cell-treated groups there was a weight gain of 0.7 ± 0.3 g and 0.4 ± 0.4 g in the combined-dose and multiple-dose groups, respectively. As a result of these contrasting patterns, the final body weight was greater in the combined-dose and multiple-doses groups than in the vehicle group (26.2 ± 0.4 g and 26.3 ± 0.3 g, respectively, vs. 23.8 ± 0.6 g, *p* < 0.001 for both, Supplementary Table S3). 

**Figure 1:**
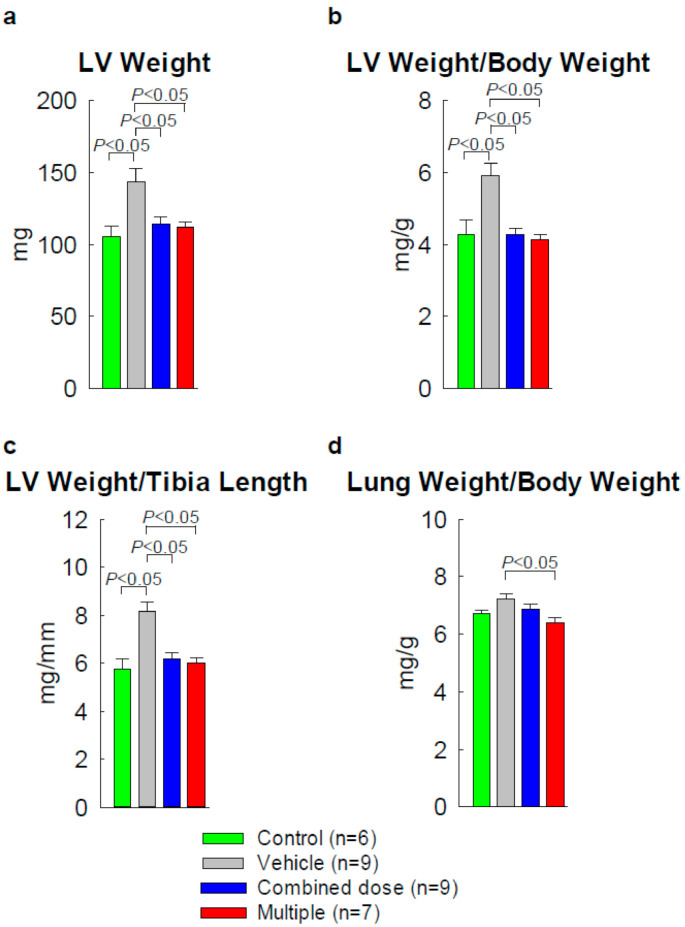
Assessment of left ventricular (LV) hypertrophy. LV weight, LV weight/body weight ratio, LV weight/tibia length ratio, and lung weight/body weight ratio. *p* < 0.05 shows statistical significance between the compared two groups. Data are means ± SEM.

We also assessed LV hypertrophy (Figure 1). In the vehicle group, LV weight, LV weight/body weight ratio, and LV weight/tibia length ratio were all significantly greater than in age-matched naive mice (+36.3%, +37.2%, and +43.8%, respectively, *p* < 0.05), indicating the occurrence of compensatory LV hypertrophy after MI (Figure 1). The LV weight/body weight ratio was significantly reduced in both cell-treated groups vs. the vehicle group (4.2 ± 0.2 and 4.2 ± 0.1 vs. 5.8 ± 0.3 mg/g, respectively, *p* < 0.05, Figure 1), indicating that cell transplantation reduced LV hypertrophy. Compared with the control group, the lung weight/body weight ratio (an index of pulmonary congestion) was significantly reduced in the multiple-dose group (6.4 ± 0.2 vs. 7.4 ± 0.4 in the vehicle group, *p* < 0.05) but not in the combined-dose group (6.9 ± 0.2) (Figure 1).

### 2.2. Effect of CPCs on LV Function Measured by Echocardiography

**Figure 2:**
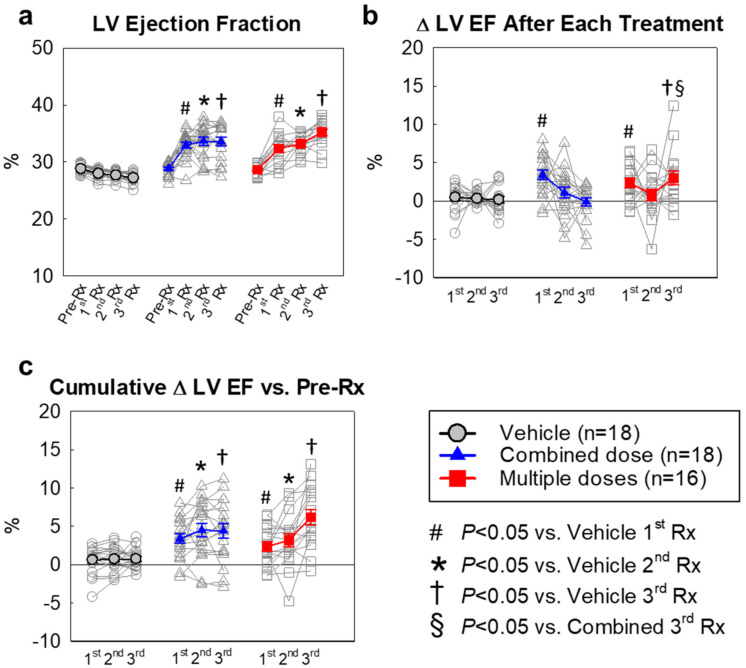
Echocardiographic assessment of left ventricular ejection fraction (LVEF). We interpreted the LVEF value in several different ways (Table 1,Table 2 and Table 3): (a) Values of LVEF in individual mice; (b) Changes in LVEF (absolute units) after the 1st, 2nd, and 3rd treatments compared with the respective pre-treatment values; (**c**) Cumulative changes in LVEF (absolute units) after the 1st, 2nd, and 3rd treatments compared with the values measured before the 1st treatment. All data are represented as the mean ± SEM.

The results of serial echocardiographic studies are summarized in Figure 2 and Supplementary Figures S1 and S2 and Table 1, Table 2 and Table 3. According to our study design, only animals with severely depressed LV function (EF ≤ 30%) at 3 months post-MI were included in the final analysis. In the vehicle group, a continuous decline in LV function was observed, with average EF decreasing from 28.8% to 27.3% in the 15-week interval between the 1st treatment and euthanasia (105 days later, Figure 2a and Table 1). 

There were no significant differences among the vehicle and cell-treated groups with respect to LVEDV, LVESV, SV, and EF at 3 months post-MI before the 1st injection (Supplementary Figure S1a–c; Figure 2), indicating that the severity of post-MI LV remodeling and dysfunction was comparable in all groups. As expected, in the vehicle group cardiac function declined after each vehicle injection. However, at 35 days after the 1st injection, both cell-treated groups exhibited a significant increase in SV compared with the vehicle group (27.3 ± 0.7 µL in combined-dose and 26.3 ± 0.5 µL in multiple-doses groups vs. 24.3 ± 0.4 µL in the vehicle group, respectively, *p* < 0.05; Table 1, Supplementary Figure S1c) and a significant decrease in ESV compared with the vehicle group (55.7 ± 1.3 µL in combined-dose and 55.2 ± 1.4 µL in multiple-doses group vs. 62.4 ± 1.0 µL in vehicle group, *p* < 0.05; Table 1, Supplementary Figure S1b). In addition, both cell-treated groups showed a significant improvement in LVEF compared with the vehicle group (33.0 ± 0.5% in the combined-dose and 32.4 ± 0.7% in the multiple-doses groups vs. 28.0 ± 0.2% in vehicle group, respectively, *p* < 0.05; Table 1, Figure 2a).

After the 2nd injection, however, the two cell-treated groups exhibited a different course with respect to LVEF and SV. In the combined-dose group, the 2nd and 3rd vehicle administrations effected no further improvement in EF and SV (Table 1, Figure 2a and Supplementary Figures S1c and S2c). Conversely, in the multiple-dose group that received three CPC infusions, the 3rd CPC injection produced a further increase in EF and SV (Table 2, Figure 2a, and Supplementary Figures S1c and S2c); as a result, in this group after the 3rd administration of CPCs, the increase in LVEF (2.0 ± 0.5%, absolute units) was significantly greater compared not only with the vehicle group (−0.5 ± 0.1%) but also with the combined-dose group (0.1 ± 0.5%; *p* < 0.05; Table 1B and Figure 2b). The total cumulative increase in EF from baseline (before the 1st injection) values (absolute EF units) was numerically greater in the multiple-dose group (6.6 ± 0.6%) than in the combined-dose group (4.8 ± 0.8%), although the difference did not achieve statistical significance (*p* = 0.087) (Table 3 and Figure 2c). 

In summary, the lower dose of CPCs (1 × 10^6^ cells) improved LV function after 35 days. This therapeutic effect at 35 days was not augmented by using a three-fold greater dose (combined dose) of CPCs (3 × 10^6^ cells) as one injection. However, a further increase in LVEF was observed when the lower dose of CPCs (1 × 10^6^ cells) was repeated two more times at 5-week intervals, particularly after the 3rd dose. At corresponding time points, no further increase in LVEF occurred in the combined-dose group, which received two doses of vehicle. 

### 2.3. Hemodynamic Measurements

**Figure 3:**
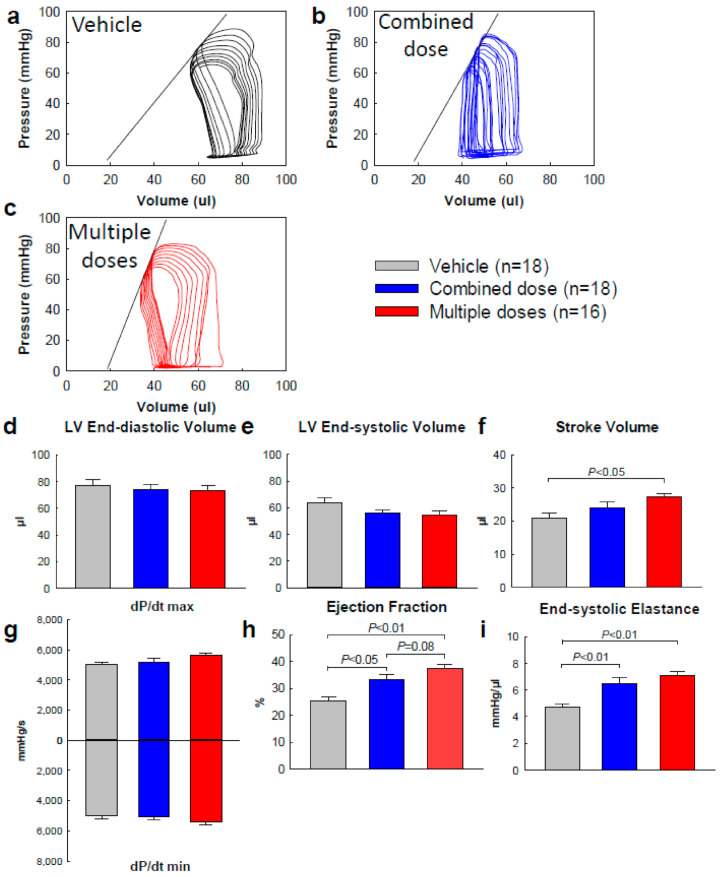
Hemodynamic assessment of global cardiac function (*n* = 16~18/group). Invasive hemodynamic studies were performed with a finest 1F Millar conductance catheter at the end of the study. (**a**–**c**) Representative pressure-volume loops recorded during preload manipulation by brief inferior vena cava occlusions. (**d**) LV end-diastolic volume. (**e**) LV end-systolic volume. (**f**) LV stroke volume. (**g**) LV pressure dP/dt maximum and dP/dt minimum. (**h**) LVEF. (**i**) LV end-systolic elastance. All data are represented as the mean ± SEM.

Cardiac function was also measured by hemodynamic studies just before euthanasia (Table 4 and Figure 3). In comparison to the vehicle group, an improvement in hemodynamic parameters was observed in both cell-treated groups, but the improvement was more pronounced in the multiple-dose group compared to the combined-dose group. Thus, compared with vehicle, LVEF was significantly increased in both groups (Table 4, Figure 3h), but was greater in the repeated-dose group; the difference had borderline statistical significance (*p* = 0.08). Stroke volume increased only in the multiple-dose group (Table 4, Figure 3f). Compared with vehicle, LV end-systolic elastance (Ees, a preload-independent functional parameter) improved in both groups, but was numerically greater in the multiple-dose group (Figure 3i, Table 4). 

In summary, two independent methods of LV functional evaluation (echocardiography and hemodynamic studies), performed by two independent investigators, demonstrated that both CPC treatments were beneficial but, in the aggregate, the benefits of three doses of 1 × 10^6^ cells were greater than those of a combined dose of 3 × 10^6^ cells.

### 2.4. Morphometric Analysis

**Figure 4:**
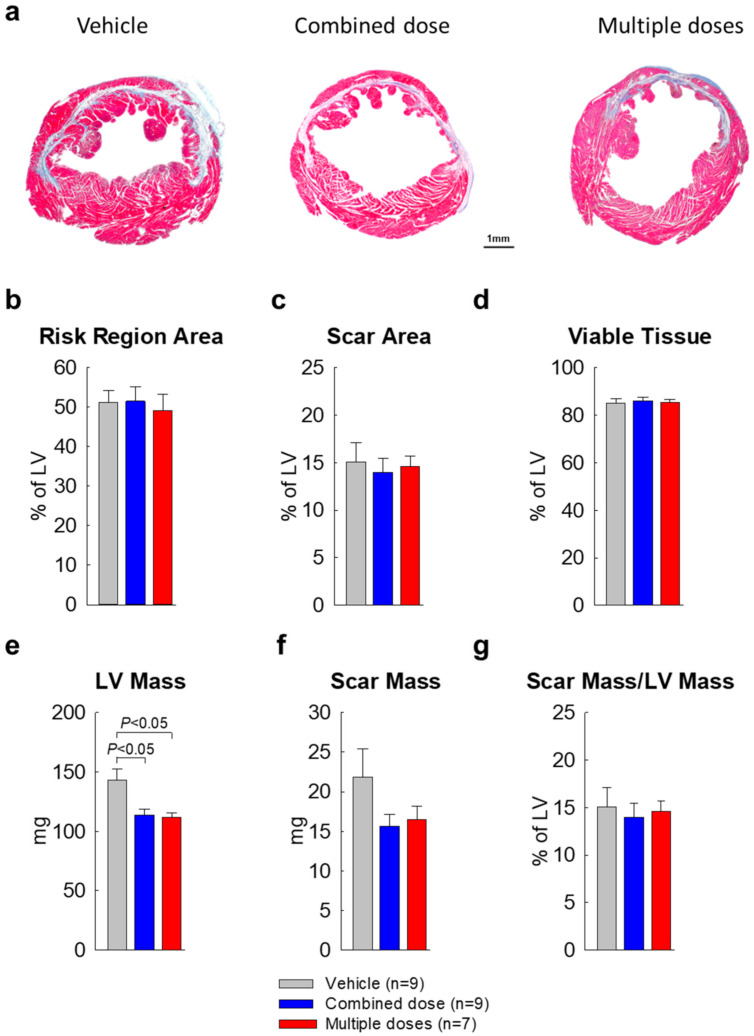
Morphological and histological assessments (*n* = 7~9/group). Masson’s trichrome–stained images were used for LV morphological measurements. (**a**) Representative Masson trichrome-stained myocardial sections. Scar tissue and viable myocardium are identified in white/blue and red, respectively; (**b**) Risk region area as percentage of LV area; (**c**) Scar area as percentage of LV area; (**d**) Viable tissue area as percentage of LV area; (**e**) LV mass; (**f**) Scar mass; (**g**) Scar mass/LV mass ratio. All data are represented as the mean ± SEM.

As described in our previous studies [5,7,11], in each heart, a detailed quantitative analysis was performed on two sections (one from each of two mid-ventricular slices); the results are summarized in Figure 4. Consistent with the post-mortem measurements, both treated groups exhibited a significantly smaller LV weight (mass) compared with the vehicle group (Figure 4e). The scar mass was numerically smaller in the two treated groups compared with vehicle, but the differences were not statistically significant (Figure 4f). The ratio of scar mass to LV mass was similar in all groups (Figure 4g). Since scar mass was not significantly different, the reduction in total LV mass in the two cell-treated groups suggests reduced LV hypertrophy.

### 2.5. LV Fibrosis

**Figure 5:**
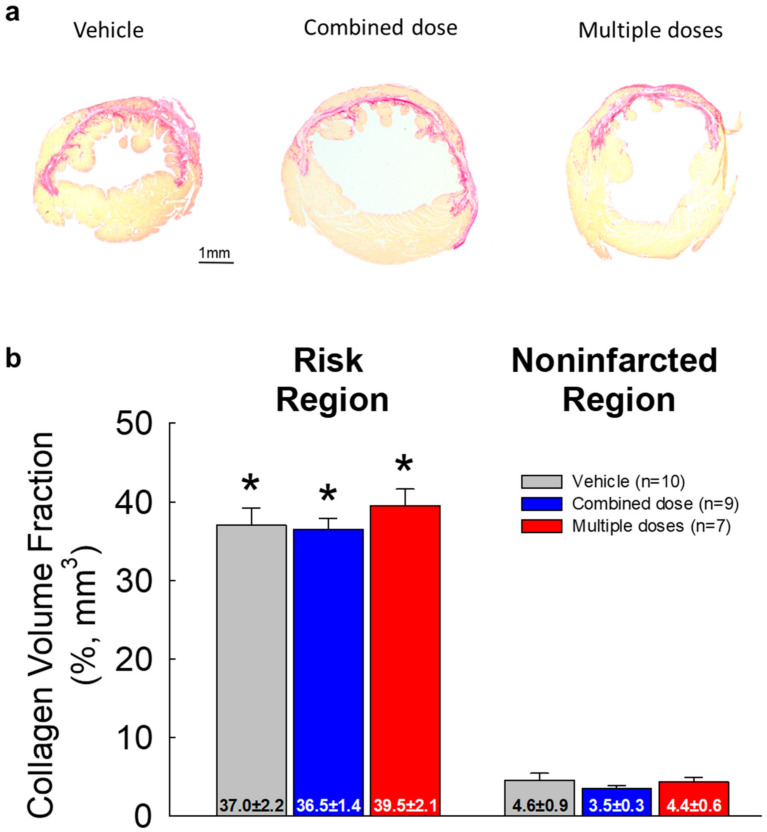
Myocardial collagen content (*n* = 7~9/group). (**a**) Representative images of LV sections stained with picrosirius red. Images were acquired under polarized light microscopy; (**b**) quantitative analysis of collagen volume fraction (mm^3^) expressed as a percentage of the risk and noninfarcted region. All data are represented as the mean ± SEM. * *p* < 0.05.

As described in our previous studies [5,7,11], quantitative analysis of collagen content was performed using picrosirius red staining and polarized light microscopy of LV sections (Figure 5). The collagen volume fraction was determined by measuring the area of stained tissue within a given field. Collagen content was expressed as a percentage of the risk region or the noninfarcted region. There was no statistically significant difference in collagen content among the three groups in either the risk region or the noninfarcted region (Figure 5, Supplementary Table S4). 

### 2.6. Cardiomyocyte Hypertrophy

**Figure 6:**
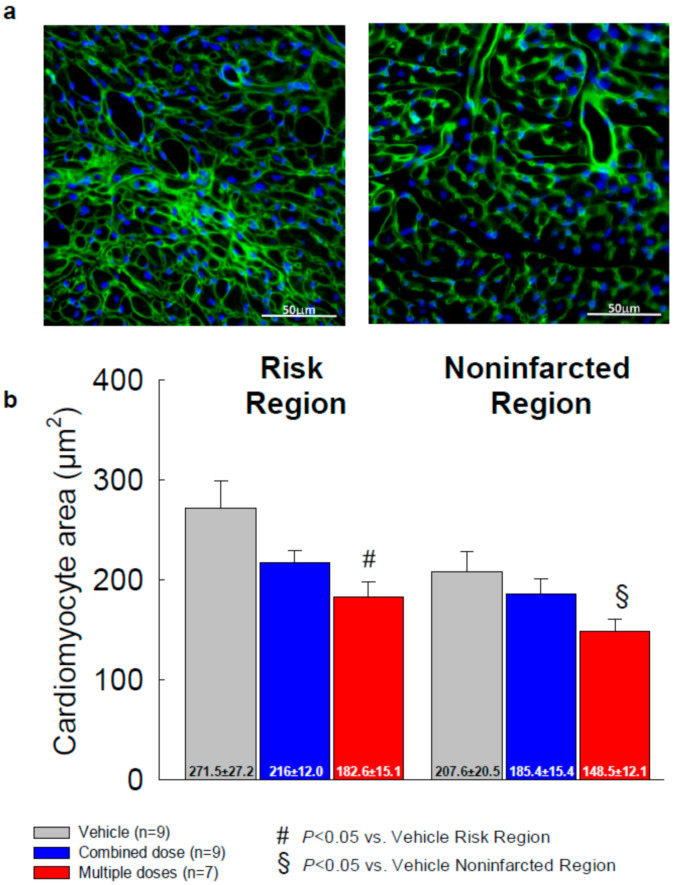
Analysis of the cross-sectional area of cardiac myocyte (*n* = 7~9/group). Myocyte cross-sectional area was assessed by immunostaining of cardiac myocytes with DAPI (blue) and WGA (green) to facilitate identification of cell membranes. (**a**) Representative microscopic images of WGA-stained LV sections; (**b**) quantitative analysis of myocyte cross-sectional area in the risk and noninfarcted regions. All data are represented as the mean ± SEM.

Chronic ischemic cardiomyopathy is associated with cardiomyocyte hypertrophy [12]. WGA staining was performed to assess whether cell therapy attenuated cardiomyocyte hypertrophy. As shown in Figure 6a, WGA demarcates the membrane of individual cells. In the combined-dose group, there was a trend towards a decrease in cardiomyocyte cross-sectional area vs. the vehicle group (−20%, 216.0 ± 12.0 µm^2^ vs. 271.5 ± 27.2 µm^2^ in the risk region and −10.7%, 185.4 ± 15.4 µm^2^ vs. 207.6 ± 20.5 µm^2^ in the noninfarcted region) but the differences did not reach statistical significance (Figure 6b). In contrast, in the multiple-dose group, cardiomyocyte area was significantly reduced both in the risk region (−32.7%, 182.6 ± 15.1 µm^2^ vs. 271.5 ± 27.2 µm^2^) and in the noninfarcted region (−28.5%, 148.5 ± 12.1 µm^2^ vs. 207.6 ± 20.5 µm^2^) compared with the vehicle group (*p* < 0.05, Figure 6b). 

### 2.7. Myocardial Content of Inflammatory Cells 

**Figure 7:**
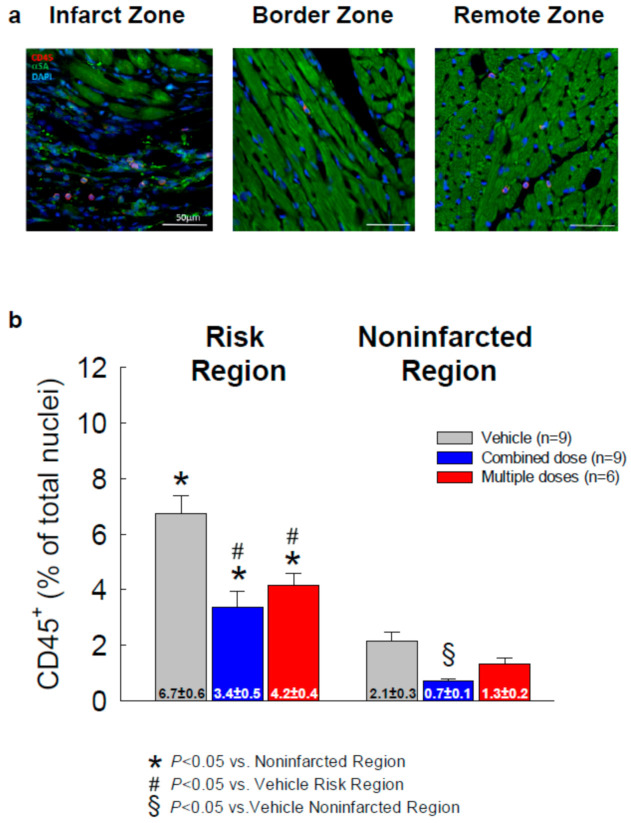
Myocardial content of inflammatory cells. (**a**) Representative microscopic images of LV sections stained for CD45 (red), α-sarcomeric actin (green), and DAPI (blue); (**b**) quantitative analysis of the number of CD45^+^ cells in the risk and noninfarcted regions expressed as a percentage of total cells counted in the corresponding region. All data are represented as the mean ± SEM.

The quantitative study of the number of CD45+ cells in the risk and noninfarcted regions was performed and expressed as a percent of total cells counted in the corresponding region (Figure 7). Predictably, in all groups, the content of CD45^+^ cells was higher in the risk region than in the noninfarcted region. Compared with the vehicle control group, the CD45^+^ cell content in the risk region was significantly lower in the combined-dose and the multiple-dose groups (3.4 ± 0.5 and 4.2 ± 0.4 vs. 6.7 ± 0.6, *p* < 0.05, respectively, Figure 7b). In the noninfarcted region, the CD45^+^ cell content was numerically lower in both cell-treated groups vs. vehicle but the difference was statistically significant only in the combined-dose group (Figure 7b). 

### 2.8. Cell Engraftment and Proliferation

No BrdU positive cells were found. IdU positive cells were infrequently observed in the scar area; these cannot be described as cardiomyocytes and were mainly interstitial cells. These data indicate that transplanted cells did not engraft to any appreciable extent in any treatment group, consistent with our previous observations [5,7,8,9].

## 3. Discussion

This study is a follow-up to our previous investigation, in which we found that administration of three doses of CPCs (1 × 10^6^ cells/dose) at 5-week intervals had significantly greater beneficial effects than a single injection of 1 × 10^6^ CPCs [7]. To determine whether these differences were caused by repeated treatments or by the higher total number of CPCs (3 × 10^6^ cells vs. 1 × 10^6^ cells), here we used the same model of chronic (3 month-old) reperfused MI and compared the effects of the same total number of CPCs (3 × 10^6^ cells) given as one injection or divided evenly into three injections (1 × 10^6^ cells). 

The results can be summarized as follows: (i) in this long-term murine model of chronic ischemic cardiomyopathy, mice in the vehicle group exhibited a progressive deterioration in LV function over the 15-week follow-up period after MI, associated with LV hypertrophy and pulmonary congestion; this syndrome simulates the clinical setting of patients with old MIs; (ii) a single combined dose of CPCs (3 × 10^6^ cells) produced a significant improvement in LV function 35 days after treatment, which persisted for at least 15 weeks; (iii) when this dose was split into three smaller doses of 1 × 10^6^ cells each, given at 5-week intervals, functional improvement was observed, particularly after the 3rd dose, such that at the end of the study, several measures of LV performance were numerically greater than in the combined-dose group, with differences in conductance catheter LVEF reaching a *p* value of 0.08; (iv) from a structural standpoint, the functional benefits imparted by both treatments were associated with a reduction in LV hypertrophy, as reflected in a reduction in LV weight, LV weight/body weight ratio, and LV weight/tibia length ratio; however, the reduction in LV hypertrophy was more pronounced in the multiple-dose group, as documented by the fact that cardiomyocyte cross-sectional area was significantly reduced only in this group; (v) the lung weight/body weight ratio (an index of pulmonary congestion) was significantly reduced only in the multiple-dose group; and (iv) the density of inflammatory cells in the infarcted region was significantly decreased in both cell-treated groups vs. the control group; in the nonischemic region, both cell-treated groups exhibited a lower density but the difference was significant only in the combined-dose group. Taken together, the results of this study support the conclusion that, although both treatments were beneficial, the effects of three CPC doses on LV function, LV hypertrophy, and pulmonary congestion cannot be fully replicated with a single combined dose. These results are consistent with our previous study in a rat model of chronic ischemic cardiomyopathy (1 month-old MI), in which we found that three repeated doses of CPCs are superior to one dose, even though the total number of cells infused was the same [8]. The consistency of our results in mouse and rat models indicates that the superiority of the repeated-treatment paradigm is not limited to one species, thereby supporting translational efforts to further test this therapeutic approach.

From a methodological standpoint, one of the distinctive features of this study was our murine model, which was designed to mimic the frequent clinical setting of patients who have coronary artery disease, suffered an MI in the distant past resulting in old scars, and now exhibit LV dysfunction secondary to chronic ischemic cardiomyopathy. At least half of clinical cases of heart failure can be ascribed to ischemic cardiomyopathy [13]. To mimic this common clinical situation, we allowed a longer interval (>3 months) between MI and treatment and an extended follow-up period (15 weeks) after treatment. These features make our murine model different from commonly used models, in which the interval between MI and study intervention is generally 4 weeks or less. Our sample sizes are relatively large (*n* = 16–18)—larger than in most murine studies. Importantly, to avoid any bias, all surgical procedures, functional measurements, and pathologic analyses were performed by investigators blinded to treatment allocation.

**Figure 8:**
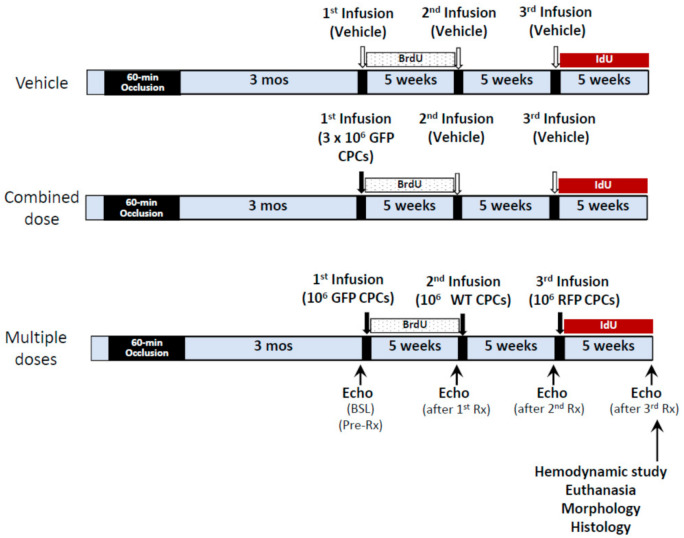
Experimental protocol. The model of chronic ischemic cardiomyopathy was created by producing an MI with a 60-min coronary artery occlusion followed a 90 days of reperfusion. Three months after MI, mice received the 1st treatment (vehicle or CPCs either 1 × 10^6^ or 3 × 10^6^) via echo-guided intraventricular injection, followed by 2nd and 3rd injections (vehicle or CPCs 1 × 10^6^) at 5-week intervals. Serial echocardiographic studies were performed before and 5 weeks after each injection. BrdU was given via mini-osmotic pumps for 5 weeks starting right after the 1st injection (until the 2nd injection). IdU was started right after the 3rd injection and continued for 5 weeks until euthanasia. Mice then underwent hemodynamic studies and the hearts were fixed for histological studies.

The conclusion that three doses are superior to a single combined dose is robust because it is based on multiple lines of evidence, both functional and structural. We evaluated cardiac function with two independent methods (echocardiography and hemodynamic studies) and assessed both load-dependent and load-independent variables. Most murine studies utilize only echocardiography and use only one parameter (e.g., LVEF or LV fractional shortening, which are load-dependent) as the read-out measure. The conclusions of the present investigation are particularly strong because they are supported by two different methods and by both load-dependent and independent measures. This concordance should give confidence that repeated cell treatments are indeed superior to a single combined treatment. The superiority of three doses is further corroborated by the structural results. Although both cell treatments reduced LV hypertrophy (Figure 2 and Figure 6), only multiple doses reduced the lung weight/body weight ratio (Figure 2), suggesting that pulmonary congestion (a hallmark of heart failure) was improved. Furthermore, the cardiomyocyte cross-sectional area (a measure of compensatory LV hypertrophy) was significantly reduced compared with vehicle hearts only in the multiple-dose group (Figure 8). Taken together, all of these diverse and independent results support the conclusion that multiple doses were more efficacious than the combined dose.

The major purpose of this investigation was to compare the efficacy of two treatment modalities. Elucidating the mechanism of benefit is beyond the scope of this study. Broadly speaking, the mechanism of action of cell therapy in general remains unclear. Our group has been the first to demonstrate, starting 10 years ago, and then has emphasized repeatedly, that CPCs do not engraft in the host heart, do not differentiate into cardiomyocytes, and therefore exert their salubrious actions via paracrine mechanisms [3,4,6,8,9,14,15,16,17,18,19] (as is the case for all other adult cells). The exact nature of these mechanisms remains unknown, not only for CPCs but also for other types of adult cells [1,3]. In the present study, staining for BrdU and IdU failed to show significant formation of new cardiomyocytes, which is consistent with our previous work [3,4,6,7,8,9,14,15,16,17,18,19]. Scar size was not reduced (Figure 4c–g). Antifibrotic actions have been postulated, but our data with picrosirius staining do not show a decrease in collagen deposition in this murine model (Figure 5). A major current hypothesis is that cell therapy imparts benefit by reducing low-level chronic inflammation [1,3]. Our data support this hypothesis: the decrease in CD45+ cells in the risk region and, to a lesser extent, the nonischemic region (Figure 7) suggests a possible anti-inflammatory action of CPCs. Other possibilities to be explored include anti-apoptotic actions, changes in myocyte contractility, or neo-angiogenesis. 

In conclusion, the present study, conducted in a clinically-relevant model and with rigorous methodology to assess cardiac function, demonstrates that administration of three cell doses improves LV function above the levels achieved with a single dose, notwithstanding the fact that the total number of cells delivered with one or three doses is the same. Thus, it is the multiplicity of doses, and not the total number of cells, that accounts for the superiority of the repeated-dose paradigm. The present study is the first to evaluate this paradigm in a model of long-term post-MI LV dysfunction. The results are consonant with our previous studies [5,7,8,9]. Together with those prior investigations, the present study supports the concept that the therapeutic effects of a cell dose in heart failure can be augmented by splitting it into multiple doses, and that the use of repeated doses is necessary to achieve the full potential of cell therapy [3]. This concept has potentially vast implications for the entire field of cell-based therapies, both at the preclinical and clinical levels. Specifically, the efficacy of cell therapy in patients with chronic ischemic cardiomyopathy may be optimized by abandoning the universal paradigm of single-dose administration and switching to repeated doses of cells [2].

## 4. Materials and Methods

This study was performed in accordance with the Guide for the Care and Use of Laboratory Animals (Department of Health and Human Services, Publication No. [NIH] 86–23) and with the guidelines of the Animal Care and Use Committee of the University of Louisville School of Medicine. Our animal proposal Number: IACUC 17084 and continuation IACUC 20805 were approved by Institutional Animal Care and Use Committee (IACUC) on 24 October 2017 and on 29 October 2020, respectively. All surgical procedures, measurements of LV function (echocardiography and hemodynamic studies), and pathological analyses were performed by investigators blinded to treatment allocation.

### 4.1. Isolation and Culture of C-kit-Positive Cardiac Cells (CPCs) 

The procedures for isolation, characterization, and culture of lin^−^/c-kit^+^ cardiac cells (CPCs) were the same as described in our previous studies [7,10]. Briefly, CPCs were isolated from wild-type, GFP and RFP transgenic male mice with same genetic background (C57BL6/J, 8–10 weeks old, purchased from the Jackson Laboratory, Bar Harbor, ME, USA). The heart was minced into ~0.5 mm^3^ of tissue pieces and cultured onto 60 mm plates to establish cell outgrowth over ~7 days using growth medium (F12 K medium supplemented with Bfgf (Sigma, St. Louis, MO, USA), LIF (Sigma, St. Louis, MO, USA), and 10% FBS (Thermo Fisher Scientific Inc., Waltham, MA, USA)). CPCs were isolated from the cell outgrowth of the explants by sequential sorting procedures, including depletion of hematopoietic lin^+^ cells (T cells, B cells, thymocytes, monocytes/macrophages, granulocytes, neutrophils, erythrocytes, and their committed bone marrow precursors) and then sorting for c-kit with a specific anti-c-kit antibody and magnetic immunobeads (Miltenyi Biotec Inc., San Diego, CA, USA). To maximize purity and positivity, the c-kit sorting procedure was repeated three times at 14-day intervals. The lin^−^/c-kit^+^ cells were cultured and the purity of the sorted cells was confirmed quantitatively by flow cytometry and immunofluorescent staining before use. In all studies, the CPCs used for cell transplantation in vivo were passaged 4–6 times after sorting [7,10].

### 4.2. Mouse Model of Ischemic Cardiomyopathy

The mouse model of myocardial ischemia and reperfusion has been described in detail in previous studies [5,7,10,15]. In this study, we performed in C57BL6/J female mice (age, 5 months; body weight, 22.7 ± 1.4 g), purchased from The Jackson Laboratory (Bar Harbor, ME, USA). Briefly, mice were anesthetized with sodium pentobarbital (Nembutal® Sodium Solution CII, Akorn Pharmaceuticals, Lake Forest, IL, USA; 60 mg/kg i.p.) and ventilated using carefully selected parameters. The chest was opened through a midline sternotomy, and a non-traumatic balloon occluder was implanted around the mid-left anterior descending coronary artery using an 8–0 nylon suture. To prevent hypotension, blood from a female donor mouse was transfused at serial times during surgery [5,7,10]. Rectal temperature was carefully monitored and maintained at 37.0 ± 0.2 °C throughout the experiment. In all groups, MI was produced by a 60-min coronary occlusion followed by reperfusion. The development of MI-induced ischemic cardiomyopathy was confirmed by a decline in ejection fraction (EF) to ≤30.0%, as measured by echocardiography [5,7,10] (Vevo 2100 Imaging System, Toronto, ON, Canada) 3 months after MI. Only mice that exhibited an EF ≤30% at 3 months after MI were included in the study.

### 4.3. Treatment Protocol

At 3 months after MI, mice were randomly allocated to three treatment groups: vehicle, single combined CPC dose, or 3 repeated CPC doses (Figure 1). All treatments (either CPCs or vehicle) were given via echo-guided injection into the LV cavity [5,7]. The 1st, 2nd, and 3rd treatments were given at 5-week intervals. Mice in the vehicle group received three doses of vehicle (phosphate-buffered saline, PBS) (200 µL for each dose); mice in the single combined CPC group received GFP^+^ CPCs (3 × 10^6^ cells in 200 µL of PBS) in the 1st injection and vehicle in the 2nd and 3rd injections; mice in the multiple-dose group received three doses of CPCs (GFP^+^ CPCs on the 1st injection, WT CPCs on the 2nd, and RPF^+^ CPCs on the 3rd; 1 × 10^6^ cells in 200 µL of PBS for each injection). To detect newly-formed cells, all mice received 5-bromo-2′-deoxyuridine (BrdU, Sigma, St. Louis, MO, USA; 33 mg/kg/day s.c.) continuously for 5 weeks starting right after the 1st treatment and 5-iodo-2′-deoxyuridine (IdU, Sigma, St. Louis, MO, USA) for 5 weeks starting right after the 3rd treatment. IdU was given in the drinking water at a final concentration of 0.1%. Neither BrdU nor IdU were applied after the 2nd dose (from week 5 to week 10). At 5 weeks after the 3rd dose, mice were subjected to final echocardiographic and hemodynamic studies and then euthanized for histologic studies (Figure 8).

### 4.4. Echo-Guided Intraventricular Injection

We used the minimally invasive delivery technique, the percutaneous, echo-guided injection to deliver the cell or vehicle into the LV cavity of mice with MI [5,7]. All injections were performed using the Vevo 2100 Imaging System (VisualSonics, Inc., Toronto, ON, Canada) equipped with a 30-MHz transducer, a Vevo Image Station with Injection Mount, and micro-manipulation controls. Mice were anesthetized with isoflurane (3% for induction, 1.5% for maintenance; Covetrus, Portland, ME, USA). The anterior chest was shaved and the animal was placed on the imaging table in the right lateral decubitus position with the left lateral side facing the injection mount. After a good long-axis view of the left ventricle was procured, the transducer was then turned clockwise 90 degrees. The left ventricle was scanned in the short-axis view from apex to base to determine the optimal site for needle insertion. To prevent bleeding from the LV wall, it is crucial to find a site that avoids the infarct scar and the coronary arteries. Under real-time B-mode view, a 0.5” length, 30 G needle attached to a 1.0 mL syringe was carefully inserted from the left lateral side and advanced into the center of the LV cavity. Successful penetration of the LV cavity was indicated by a small reflux of bright red blood from the needle into the syringe tip. CPCs or PBS were infused at a steady rate over 100 s. After the infusion, the needle was quickly withdrawn from the left ventricle. The body temperature of the animal was controlled in the range of 37 ± 0.2 °C, and the electrocardiogram and respiration were monitored carefully during the whole procedure. Mice were allowed to recover in temperature-controlled and oxygen-rich tents as needed.

### 4.5. Echocardiographic Studies

In order to detect the cardiac function repeatedly in vivo, non-invasive echocardiographic studies were performed using the Vevo 2100 Imaging System (VisualSonics, Inc., Toronto, ON, Canada) equipped with a 30-MHz transducer as previous studies [5,7,10]. Serial echocardiograms were obtained before treatment (3 months after MI, before the 1st injection) and 5 weeks after each treatment and were performed under isoflurane anesthesia (3% for induction, 1% for maintenance). Using a rectal temperature probe, body temperature was carefully maintained at 37 ± 0.2 throughout the study. The parasternal long-axis and parasternal short-axis views were used to obtain 2D mode images for the measurement of LV mass, end-diastolic and end-systolic LV volume (LVEDV and LVESV), stroke volume (SV), and EF, as previously described [7,10]. Digital images were analyzed off-line by blinded observers using the Vevo 2100 workstation software (VisualSonics, Inc., Toronto, ON, Canada). Measurements were performed according to the American Society for Echocardiography. At least three measurements were taken and averaged for each parameter. 

### 4.6. Hemodynamic Studies

Hemodynamic studies were performed at 5 weeks after the 3rd injection, just before euthanasia, as previously described [5,7]. Mice were anesthetized with sodium pentobarbital (60 mg/kg i.p.), intubated, and ventilated with a positive pressure ventilator (Hugo-Sachs Electronik D-79232 [Germany]; ventilation rate, 105/min; tidal volume 10.3 µL/g). Rectal temperature was kept at 37 ± 0.2 °C. A 1.0 French pressure-volume (PV) catheter (PVR-1035, Millar Instruments, Houston, Texas) was inserted into the left ventricle via the right carotid artery. The position of the catheter was carefully adjusted until typical and stable PV loop signals were acquired. After 30 min of stabilization, the PV signals were recorded continuously with an MPVS ULTRA Pressure-Volume Unit (Millar Instruments, Houston, TX, USA) coupled with a Powerlab 16/30 converter (AD Instruments, Colorado Springs, CO, USA), stored, and displayed on a computer with Lab Chart 7.0 software (AD Instruments, Colorado Springs, CO, USA). Inferior vena cava occlusions were performed with external compression to produce variably loaded beats for determination of the end-systolic PV relation and other derived constructs of LV performance. Parallel conductance from surrounding structures was calculated by a bolus injection of 5 µL of 30% NaCl through the jugular vein. Echocardiography-derived SV was used as outside reference in alpha calibration for LV volume. All hemodynamic data analyses were performed off-line using Lab Chart 7.0 software (AD Instruments, Colorado Springs, CO, USA) by an investigator blinded to the treatment.

### 4.7. Histological Studies

The protocol for histologic analysis has been described [5,7,11]. Briefly, at the end of the hemodynamic study, the heart was arrested in diastole by an i.v. injection of 0.15 mL of CdCl_2_ (100 mM, Millipore-Sigma, St Louis, MO, USA), excised and perfused retrogradely at 60–80 mmHg (LVEDP = 8 mmHg) with heparinized PBS followed by 10% neutral buffered formalin solution for 15 min. The heart was fixed in formalin for 24 h, then sectioned into three transverse slices (~2 mm thick) from apex to base, and subjected to tissue processing and paraffin embedding. Slices were sectioned at 4-μm intervals and stained with Masson’s trichrome (Millipore-Sigma, St Louis, MO, USA), picrosirius red, or antibodies against EGFP and cell-type-specific markers. Images were acquired digitally with Nikon Ni-E and Nikon TiE light microscopes and analyzed using Nikon Elements software [NIS, 64-bit version 3.22.00 (Build 710), Nikon Inc., Melville, NY, USA]. From the Masson’s trichrome–stained images, morphometric parameters, including total LV area, risk region area, and scar area, were measured in each section [5]. Myocardial collagen content was quantitated on picrosirius red-stained heart images acquired under polarized light microscopy by determining collagen density (arbitrary unit) per mm^2^ of risk region or non-infarcted region with the NIS Elements software [5,7,11]. Collagen content was expressed as collagen volume fraction (CVF) by dividing the weight of the collagen fraction by the density of the myocardium (1.055). Fluorescent stains and antibodies were used to identify specific cells, cell markers, and compartments. Imaging was performed using Nikon Ni-E and Nikon TiE light microscopes; digitally acquired images were analyzed using NIS-Elements software. 

### 4.8. Immunohistochemistry

Immunohistochemistry was performed in formalin-fixed, paraffin-embedded, 4-μm-thick heart sections as described previously [5,7,8,11]. Myocytes were stained with an anti-α-sarcomeric actin (α-SA) antibody (Millipore-Sigma, St Louis, MO, USA). Myocyte membranes were stained with Rhodamine conjugated wheat germ agglutinin (WGA) (Vector Laboratories, Inc., Burlingame, CA, USA) to facilitate the identification of individual myocytes for analysis of myocyte cross-sectional area and myocyte density by identification of cell borders. Double immunofluorescent staining was performed with specific anti- α-SA and anti-BrdU or anti-IdU (Abcam, Cambridge, MA, USA) antibodies for evaluation of proliferating myocytes. An anti-CD45 (Abcam, Cambridge, MA, USA) antibody was used to identify inflammatory cells. Nuclei were stained with DAPI (4′,6-diamidino-2-phenylindole).

### 4.9. Statistical Analysis

Data are presented as mean ± SEM. Data were tested for normality using the Shapiro-Wilk test. Data that are normally distributed were analyzed with one-way or two-way ANOVA, followed by unpaired Student’s *t*-tests with the Bonferroni correction [20]. The Kruskal–Wallis one-way ANOVA on ranks was used for data that are not normally distributed. A *p* value <0.05 was considered statistically significant. All statistical analyses were performed using the Sigma Stat software system (San Jose, CA, USA) [5,7].

## Figures and Tables

**Table 1:** Echocardiographic Results.

	*n*=	Before 1st Rx	Before 2nd Rx	Before 3rd Rx	Final
**EDV (µL)**					
Vehicle	18	82.6± 1.4	86.7 ± 1.4	88.8 ± 1.4	90.8 ± 1.8
Combined dose	18	83.0 ± 1.5	83.0 ± 1.7	84.7 ± 1.7	84.6 ± 1.6 ^#^
Multiple doses	16	83.4 ± 1.9	81.5 ± 1.5	81.9 ± 2.2 ^#^	85.4 ± 1.1
**ESV (µL)**					
Vehicle	18	58.8 ± 1.0	62.4 ± 0.8	64.2 ± 1.0	66.1 ± 1.3
Combined dose	18	59.0 ± 1.0	55.7 ± 1.3 ^#^	56.3 ± 1.4 ^#^	56.2 ± 1.4 ^#^
Multiple doses	16	59.5 ± 1.4	55.2 ± 1.4 ^#^	54.8 ± 1.7 ^#^	55.3 ± 0.9 ^#^
**SV(µL)**					
Vehicle	18	23.8 ± 0.4	24.3 ± 0.4	24.6 ± 0.4	24.7 ± 0.5
Combined dose	18	23.9 ± 0.5	27.3 ± 0.7 ^#^	28.4 ± 0.7 ^#^	28.0 ± 0.7 ^#^
Multiple doses	16	23.9 ± 0.6	26.3 ± 0.5 ^#^	27.1 ± 0.6 ^#^	30.4 ± 0.6 ^#^
**EF (%)**					
Vehicle	18	28.8 ± 0.2	28.0 ± 0.2	27.7 ± 0.2	27.3 ± 0.2
Combined dose	18	28.9 ± 0.3	33.0 ± 0.5 ^#^	33.7 ± 0.7 ^#^	33.6 ± 0.8 ^#^
Multiple doses	16	28.7 ± 0.2	32.4 ± 0.7 ^#^	33.2 ± 0.4 ^#^	35.2 ± 0.6 ^#^

Rx indicates treatment; EDV, end-diastolic volume; ESV, end-systolic volume; SV, stroke volume; EF, ejection fraction. To distinguish from the group assignment, these four major parameters EDV, ESV, SV, and EF are bold and underlined. We present the mean values of these major parameters in this table. ^#^ *p* < 0.05 vs. Vehicle. All data are represented as the mean ± SEM.

**Table 2:** Change in Echocardiographic Parameters after each Treatment.

	*n*=	After 1st Rx	After 2nd Rx	After 3rd Rx
**Δ EDV (µL)**				
Vehicle	18	4.0 ± 1.4	2.1± 0.6	2.1 ± 1.4
Combined dose	18	−0.1 ± 1.5	1.7 ± 1.7	−0.2 ± 1.6
Multiple doses	16	−2.1 ± 1.7 ^#^	0.6 ± 2.2	3.5 ± 2.0
**Δ ESV (µL)**				
Vehicle	18	3.6 ± 1.0	1.8 ± 0.4	1.9 ± 1.0
Combined dose	18	−3.3 ± 1.0 ^#^	0.6 ± 1.4	−0.1 ± 1.3
Multiple doses	16	−4.3 ± 1.3 ^#^	−0.4 ± 1.8	0.5 ± 1.4
**Δ SV (µL)**				
Vehicle	18	0.5 ± 0.4	0.3 ± 0.2	0.1 ± 0.4
Combined dose	18	3.4 ± 0.6 ^#^	1.1 ± 0.7	−0.1 ± 0.5
Multiple doses	16	2.4 ± 0.6 ^#^	0.8 ± 0.8	3.0 ± 0.9 ^†,§^
**Δ EF (%)**				
Vehicle	18	−0.8 ± 0.2	−0.3 ± 0.1	−0.5 ± 0.1
Combined dose	18	4.1 ± 0.5 ^#^	0.7 ± 0.8	0.1 ± 0.5
Multiple doses	16	3.7 ± 0.6 ^#^	0.8 ± 0.6	2.0 ± 0.5 ^†,§^

Rx indicates treatment; EDV, end-diastolic volume; ESV, end-systolic volume; EF, ejection fraction. To distinguish from the group assignment, these four major parameters EDV, ESV, SV, and EF are bold and underlined. We present the absolute value of the changes after each treatment of these four major underlined parameters in this table. ^#^ *p* < 0.05 vs. Vehicle 1st Rx, ^†^ *p* < 0.05 vs. Vehicle 3rd Rx, ^§^ *p* < 0.05 vs. Combined dose 3rd Rx. All data are represented as the mean ± SEM.

**Table 3:** Cumulative Change in Echocardiographic Parameters after each Treatment.

	*n*=	After 1st Rx	After 2nd Rx	After 3rd Rx
**Δ EDV (µL)**				
Vehicle	18	4.0 ± 1.4	6.1 ± 1.1	8.2 ± 1.1
Combined dose	18	−0.1 ± 1.5	1.8 ± 1.6	1.6 ± 1.9 ^†^
Multiple doses	16	−2.1 ± 1.7 ^#^	−1.6 ± 3.0 *	2.0 ± 2.3
**Δ ESV (µL)**				
Vehicle	18	3.6 ± 1.0	5.4 ± 0.8	7.3 ± 0.9
Combined dose	18	−3.3 ± 1.0 ^#^	−2.7 ± 1.1 *	−2.8 ± 1.4 ^†^
Multiple doses	16	−4.3 ± 1.3 ^#^	−4.7 ± 2.2 *	−4.2 ± 1.6 ^†^
**Δ SV (µL)**				
Vehicle	18	0.5 ± 0.4	0.8 ± 0.4	0.9 ± 0.3
Combined dose	18	3.4 ± 0.6 ^#^	4.5 ± 0.9 *	4.4 ± 1.0 ^†^
Multiple doses	16	2.4 ± 0.6	3.2 ± 0.9 *	6.2 ± 1.0 ^†^
**Δ EF (%)**				
Vehicle	18	−0.8 ± 0.2	−1.1 ± 0.2	−1.6 ± 0.2
Combined dose	18	4.1 ± 0.5 ^#^	4.8 ± 0.8 *	4.8 ± 0.8 ^†^
Multiple doses	16	3.7 ± 0.6 ^#^	4.5 ± 0.4 *	6.6 ± 0.6 ^†^

Rx indicates treatment; EDV, end-diastolic volume; ESV, end-systolic volume; EF, ejection fraction. To distinguish from the group assignment, these four major parameters EDV, ESV, SV, and EF are bold and underlined. The total cumulative values of these parameters are presented in this table. ^#^ *p* < 0.05 vs. Vehicle 1st Rx, * *p* < 0.05 vs. Vehicle 2nd Rx, ^†^ *p* < 0.05 vs. Vehicle 3rd Rx. All data are represented as the mean ± SEM.

**Table 4:** Hemodynamics Results.

	Vehicle (*n* = 18)	Combined Dose (*n* = 18)	Multiple Doses (*n* = 16)
EDV (µL)	77.3 ± 4.1	74.1 ± 3.9	73.3 ± 4.0
ESV (µL)	63.7 ± 3.5	56.3 ± 2.3	54.4 ± 3.2
SV (µL)	20.0 ± 1.5	24.5 ± 2.0	27.1 ± 1.3 ^#^
+dP/dt (mmHg/s)	5003 ± 177	5192 ± 239	5620 ± 169
−dP/dt (mmHg/s)	−4997 ± 209	−5087 ± 170	−5427 ± 166
EF (%)	25.7 ± 1.2	33.2 ± 1.7 ^#^	37.4 ± 1.5 ^#^
Ees (mmHg/µL)	4.8 ± 0.2	6.5 ± 0.5 ^#^	7.1 ± 0.3 ^#^

EDV indicates end-diastolic volume; ESV, end-systolic volume; SV, stroke volume; ±dP/dt, maximum and minimum rate of pressure change; EF, ejection fraction; Ees, end-systolic elastance. ^#^ *p* < 0.05 vs. Vehicle. All data are represented as the mean ± SEM.

## Data Availability

Not applicable.

## Supplementary Materials

The following are available online at https://www.mdpi.com/1422-0067/22/6/3145/s1.

## Abbreviations

CPC	c-kit-positive cardiac cell
CMC	Cardiac mesenchymal cell
MI	Myocardial infarction
LV	Left ventricle
LVEDV	End-diastolic LV volume
LVESV	End-systolic LV volume
SV	Stroke volume
EF	Ejection fraction
PV	Pressure–volume
WGA	Wheat germ agglutinin

## References

[1] Bolli R., Kahlon A. (2020). Time to end the war on cell therapy. Eur. J. Heart Fail..

[2] Bolli R. (2017). Repeated Cell Therapy: A Paradigm Shift Whose Time Has Come. Circ. Res..

[3] Wysoczynski M., Khan A., Bolli R. (2018). New Paradigms in Cell Therapy: Repeated Dosing, Intravenous Delivery, Immunomodulatory Actions, and New Cell Types. Circ. Res..

[4] Hong K.U., Guo Y., Li Q.-H., Cao P., Al-Maqtari T., Vajravelu B.N., Du J., Book M.J., Zhu X., Nong Y. (2014). c-kit+ Cardiac Stem Cells Alleviate Post-Myocardial Infarction Left Ventricular Dysfunction Despite Poor Engraftment and Negligible Retention in the Recipient Heart. PLoS ONE.

[5] Guo Y., Wysoczynski M., Nong Y., Tomlin A., Zhu X., Gumpert A.M., Nasr M., Muthusamy S., Li H., Book M. (2017). Repeated doses of cardiac mesenchymal cells are therapeutically superior to a single dose in mice with old myocardial infarction. Basic Res. Cardiol..

[6] Tang X.L., Li Q., Rokosh G., Sanganalmath S.K., Chen N., Ou Q., Stowers H., Hunt G., Bolli R. (2016). Long-Term Outcome of Administration of c-kit(POS) Cardiac Progenitor Cells After Acute Myocardial Infarction: Transplanted Cells Do not Become Cardiomyocytes, but Structural and Functional Improvement and Proliferation of Endogenous Cells Persist for at Least One Year. Circ. Res..

[7] Guo Y., Nong Y., Li Q., Tomlin A., Kahlon A., Gumpert A., Slezak J., Zhu X., Bolli R. (2020). Comparison of One and Three Intraventricular Injections of Cardiac Progenitor Cells in a Murine Model of Chronic Ischemic Cardiomyopathy. Stem Cell Rev. Rep..

[8] Tang X., Nakamura S., Li Q., Wysoczynski M., Gumpert A.M., Wu W., Hunt G., Stowers H., Ou Q., Bolli R. (2018). Repeated Administrations of Cardiac Progenitor Cells Are Superior to a Single Administration of an Equivalent Cumulative Dose. J. Am. Hear. Assoc..

[9] Tokita Y., Tang X.L., Li Q., Wysoczynski M., Hong K.U., Nakamura S., Wu W.J., Xie W., Li D., Hunt G. (2016). Repeated Administrations of Cardiac Progenitor Cells Are Markedly More Effective Than a Single Administration: A New Paradigm in Cell Therapy. Circ. Res..

[10] Li Q., Guo Y., Ou Q., Chen N., Wu W.-J., Yuan F., O’Brien E., Wang T., Luo L., Hunt G.N. (2011). Intracoronary administration of cardiac stem cells in mice: A new, improved technique for cell therapy in murine models. Basic Res. Cardiol..

[11] Wysoczynski M., Guo Y., Moore J.B., Muthusamy S., Li Q., Nasr M., Li H., Nong Y., Wu W., Tomlin A.A. (2017). Myocardial Reparative Properties of Cardiac Mesenchymal Cells Isolated on the Basis of Adherence. J. Am. Coll. Cardiol..

[12] Pfeffer M.A., Braunwald E. (1990). Ventricular remodeling after myocardial infarction. Experimental observations and clinical implications. Circulation.

[13] Benjamin E.J., Muntner P., Alonso A., Bittencourt M.S., Callaway C.W., Carson A.P., Chamberlain A.M., Chang A.R., Cheng S., Das S.R. (2019). Heart Disease and Stroke Statistics-2019 Update: A Report from the American Heart Association. Circulation.

[14] Bolli R., Tang X.-L., Sanganalmath S.K., Rimoldi O., Mosna F., Abdel-Latif A., Jneid H., Rota M., Leri A., Kajstura J. (2013). Intracoronary Delivery of Autologous Cardiac Stem Cells Improves Cardiac Function in a Porcine Model of Chronic Ischemic Cardiomyopathy. Circulation.

[15] Hong K.U., Li Q.-H., Guo Y., Patton N.S., Moktar A., Bhatnagar A., Bolli R. (2013). A highly sensitive and accurate method to quantify absolute numbers of c-kit+ cardiac stem cells following transplantation in mice. Basic Res. Cardiol..

[16] Keith M.C., Bolli R. (2015). “String theory” of c-kit(pos) cardiac cells: A new paradigm regarding the nature of these cells that may reconcile apparently discrepant results. Circ Res.

[17] Sanganalmath S.K., Bolli R. (2013). Cell therapy for heart failure: A comprehensive overview of experimental and clinical studies, current challenges, and future directions. Circ. Res..

[18] Tang X.-L., Rokosh G., Sanganalmath S.K., Tokita Y., Keith M.C.L., Shirk G., Stowers H., Hunt G.N., Wu W., Dawn B. (2015). Effects of Intracoronary Infusion of Escalating Doses of Cardiac Stem Cells in Rats With Acute Myocardial Infarction. Circ. Hear. Fail..

[19] Bolli R., Ghafghazi S. (2017). Stem cells: Cell therapy for cardiac repair: What is needed to move forward?. Nat. Rev. Cardiol..

[20] Guo Y., Bao W., Wu W.-J., Shinmura K., Tang X.-L., Bolli R. (2000). Evidence for an essential role of cyclooxygenase-2 as a mediator of the late phase of ischemic preconditioning in mice. Basic Res. Cardiol..

