# Supplementary material for: Comparison of Repeated Doses of C-kit-Positive Cardiac Cells versus a Single Equivalent Combined Dose in a Murine Model of Chronic Ischemic Cardiomyopathy
Source: Int J Mol Sci. 2021 Mar 19;22(6):3145. doi: 10.3390/ijms22063145 (PMC8003463; doi:10.3390/ijms22063145)

Suppl. Figure 1

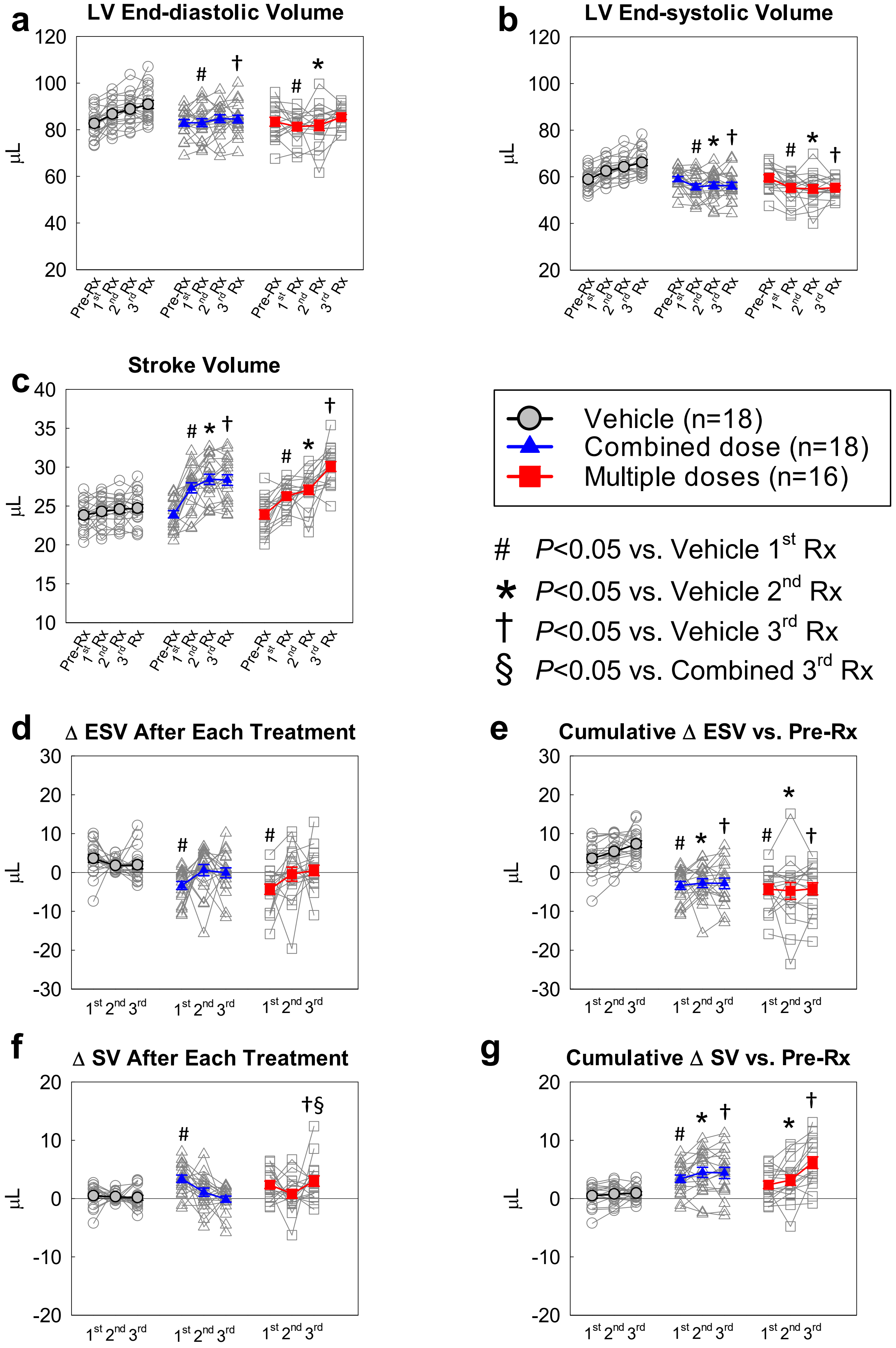

Suppl. Figure 2

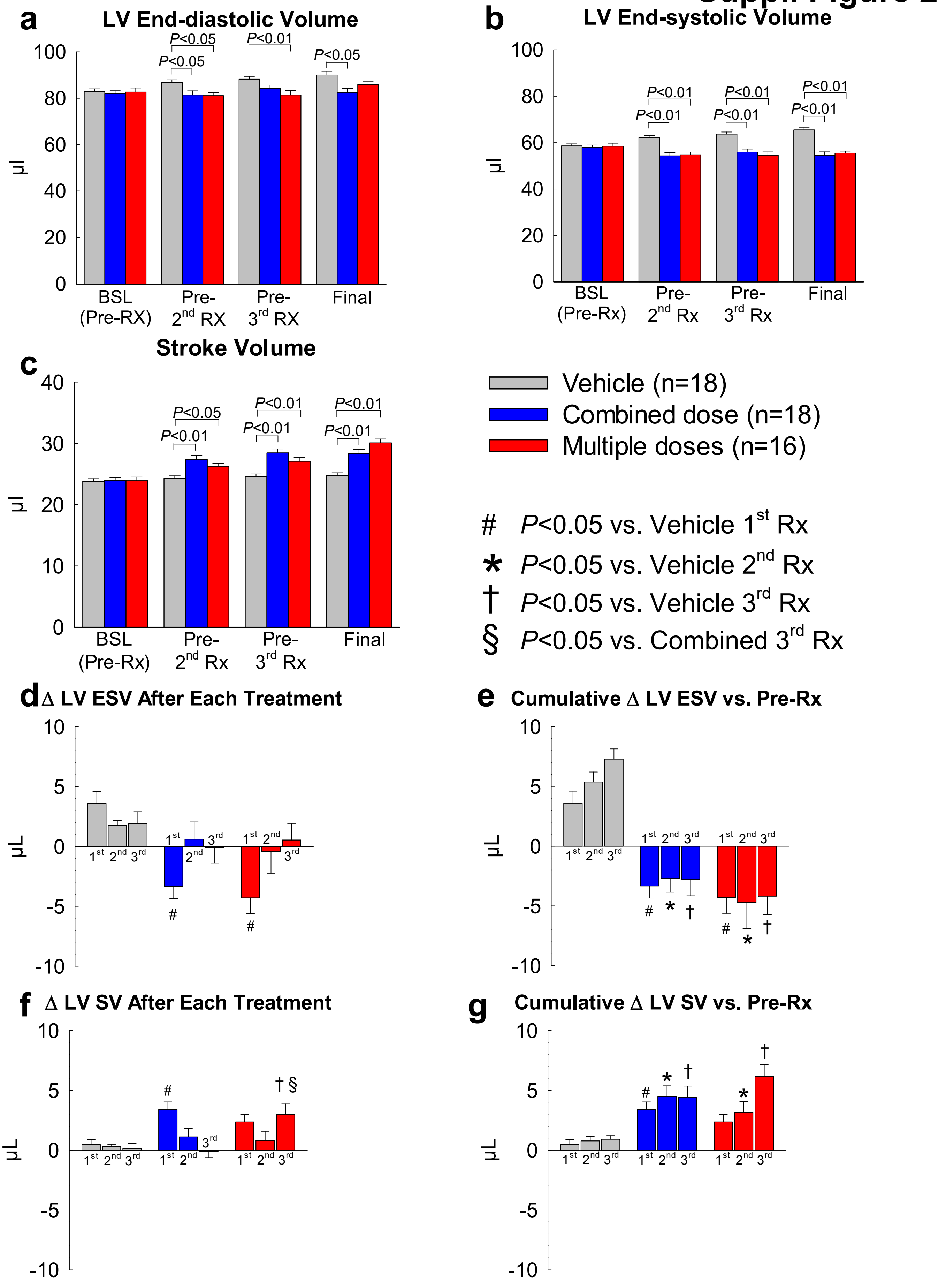

Supplement: Supplementary file 1 [file ijms-22-03145-s001.zip › Suppl final.pdf]
