# Supplementary material for: Comparison of Repeated Doses of C-kit-Positive Cardiac Cells versus a Single Equivalent Combined Dose in a Murine Model of Chronic Ischemic Cardiomyopathy
Source: Int J Mol Sci. 2021 Mar 19;22(6):3145. doi: 10.3390/ijms22063145 (PMC8003463; doi:10.3390/ijms22063145)
Supplement: Supplementary file 1 [file ijms-22-03145-s001.zip › Supplementary Figures legend R1.pdf]

Supplementary Figures 1 & 2. Echocardiographic assessment of LV volumes. The same results are presented in two different ways, dot plot (Suppl. Fig. 1) and bar graph (Suppl. Fig. 2). The acquisitions of the serials of echocardiographic images were performed before each treatment and at the end of the study. **a**, LV end-diastolic volume; **b**, LV end-systolic volume; **c**, LV stroke volume; d, Changes in LV ESV after the 1st, 2nd, and 3rd treatments compared with the pre-treatment values; **e**, Cumulative changes in LV ESV after the 1st, 2nd, and 3rd treatments compared with the values measured before the 1st treatment; **f,** Changes in LV SV after the 1st, 2nd, and 3rd treatments compared with the pre-treatment values; **g**, Cumulative changes in LV SV after the 1st, 2nd, and 3rd treatments com-pared with the values measured before the 1st treatment. All data are represented as the mean ± SEM.
