# Supplementary material for: Comparison of Repeated Doses of C-kit-Positive Cardiac Cells versus a Single Equivalent Combined Dose in a Murine Model of Chronic Ischemic Cardiomyopathy
Source: Int J Mol Sci. 2021 Mar 19;22(6):3145. doi: 10.3390/ijms22063145 (PMC8003463; doi:10.3390/ijms22063145)
Supplement: Supplementary file 1 [file ijms-22-03145-s001.zip › Tables S1-4 final.pdf]

Supplementary Table 1. Exclusions

	Vehicle	Combined-dose	Multiple-doses
**Initial enrollment of mice for MI studies**	110		
**Death after MI (all deaths were within 48 h)**	21		
**Excluded because EF >30%**	29		
**Mice assigned to group**	18	20	22
**Death after 1st infusion**	0	2	3
**Death after 2nd infusion**	0	0	2
**Death after 3rd infusion**	0	0	1
**Total mice included in final analysis**	18	18	16

EF, indicates ejection fraction; MI, indicates myocardial infarction.

Supplementary Table 2.
A. Rectal Temperature and Heart Rate on the Day of Coronary Occlusion

	Pre-	Occlusion			Reperfusion		
		10 min	30 min	60 min	1 min	5 min	10 min
**Temperature (°C)**							
Vehicle	37.1 ± 0.0	37.0 ± 0.0	37.0 ± 0.0	37.1 ± 0.0	37.1 ± 0.0	37.0 ± 0.0	37.1 ± 0.1
Combined dose	37.1 ± 0.0	37.0 ± 0.0	37.1 ± 0.0	37.1 ± 0.0	37.1 ± 0.0	37.0 ± 0.0	37.1 ± 0.0
Multiple doses	37.1 ± 0.0	37.1 ± 0.1	37.1 ± 0.0	37.1 ± 0.0	37.1 ± 0.0	37.1 ± 0.1	37.1 ± 0.0
**Heart rate (beats/min)**							
Vehicle	466 ± 10	451 ± 7	464 ± 11	496 ± 13	508 ± 10	532 ± 14	546 ± 14
Combined dose	452 ± 12	465 ± 16	474 ± 12	479 ± 8	517 ± 20	501 ± 11	502 ± 12
Multiple doses	455 ± 8	449 ± 7	476 ± 12	498 ± 11	503 ± 6	509 ± 9	508 ± 17

A. Measurements of rectal temperature and heart rate were taken before the 60-min coronary occlusion (pre-occlusion), at 10, 30, and 60 min into the 60-min occlusion, and at 1, 5, and 10 min after reperfusion. Rectal temperature was continuously monitored and carefully controlled throughout the experiment, as detailed in the text. Data are means ± SEM.

B. Rectal Temperature and Heart Rate on the Days of the Echocardiogram

	Pre-Rx 1 (BSL)	Pre-Rx 2	Pre-Rx 3	Final Echo
**Temperature (°C)**				
Vehicle	37.4 ± 0.1	37.7 ± 0.1	37.7 ± 0.1	37.6 ± 0.1
Combined dose	37.5 ± 0.1	37.8 ± 0.1	37.5 ± 0.1	37.7 ± 0.1
Multiple doses	37.3 ± 0.1	37.6 ± 0.1	37.6 ± 0.1	37.8 ± 0.2
**Heart rate (beats/min)**				
Vehicle	522 ± 7	522 ± 11	550 ± 9	494 ± 10
Combined dose	516 ± 11	535 ± 9	551 ± 11	503 ± 12
Multiple doses	506 ± 7	534 ± 14	547 ± 7	523 ± 12

B. Measurements of rectal temperature and heart rate were taken during the echocardiographic studies. First, second, and third Rx indicates first, second, and third treatment. Data are means ± SEM.

C. Temperature and Heart Rate on the Day of the Hemodynamics Study

	Temperature (°C)	Heart rate (beats/min)
Vehicle	37.2 ± 0.03	466 ± 10
Combined dose	37.3 ± 0.03	460 ± 12
Multiple doses	37.2 ± 0.02	476 ± 14

C. Measurements of rectal temperature and heart rate were taken after LV catheterization was stabilized for 30 minutes during hemodynamics studies. Data are means ± SEM.

Supplementary Table 3.
Body Weight on the Days of Coronary Occlusion and Echocardiograms

	Naive (n=6)	Vehicle (n=18)	Combined dose (n=18)	Multiple doses (n=16)
**Body weight(g)**				
Day of Occlusion		21.8 ± 0.2	23.3 ± 0.4#	23.2 ± 0.3#
Pre-Rx 1 (BSL)		25.3 ± 0.4	25.5 ± 0.4	25.9 ± 0.5
Pre-Rx 2		25.3 ± 0.4	25.9 ± 0.3	26.2 ± 0.4
Pre-Rx 3		25.6 ± 0.5	26.1 ± 0.4	25.2 ± 0.4
Final Echo	24.8 ± 0.7	23.8 ± 0.6	26.2 ± 0.4#	26.3 ± 0.3#
**Δ Body Weight(g)**				
Final-Pre-Rx 1		-1.5 ± 0.8	0.7 ± 0.3#	0.4 ± 0.4
Final-Pre-Rx 2		-1.5 ± 0.6	0.3 ± 0.2#	0.3 ± 0.4#
Final-Pre-Rx 3		-1.8 ± 0.6	0.1 ± 0.4#	1.1 ± 0.4#

Measurements of body weight taken on the day of 60-minute coronary occlusion and on the day of each echocardiographic study. Echocardiograms were performed prior to the first treatment (3 months after 60-minute coronary occlusion), and 5 weeks after each treatment. Rx indicates treatment. # $P<0.05$ vs. Vehicle. Data are means ± SEM.

Supplementary Table 4.
Collagen in Left Ventricle

	**Vehicle (n=11)**	**Combined dose (n=11)**	**Multiple doses (n=10)**
CV (mm^3) in Risk Region	16.7 ± 1.6*	16.2 ± 1.3*	15.4 ± 1.8*
CV (mm^3) in Noninfarcted Region	4.4 ± 1.0	2.3 ± 0.8	2.6 ± 0.3
CVF (%) in Risk Region	37.3 ± 2.0*	37.2 ± 1.4*	37.2 ± 1.9*
CVF (%) in Noninfarcted Region	4.9 ± 0.9	3.6 ± 0.3	4.4 ± 0.4

Comparison of collagen volume and collagen volume fraction among 65-days post-MI with/without cell therapy. CV: Collagen Volume; CVF: Collagen Volume Fraction. *$P<0.05$ vs. Noninfarcted Region. Data are means ± SEM.
